# Monolithic III–V membrane photonic crystal lasers on SOI using selective lateral heteroepitaxy

**DOI:** 10.1038/s41377-025-02074-8

**Published:** 2026-01-30

**Authors:** Cong Zeng, Zhaojie Ren, Zili Lei, Donghui Fu, Yingzhi Zhao, Ying Yu, Yu Han, Siyuan Yu

**Affiliations:** State Key Laboratory of Optoelectronic Materials and Technologies, School of Electronics and Information Technology, Sun Yat-sen University, Guangzhou, China

**Keywords:** Photonic crystals, Silicon photonics, Semiconductor lasers

## Abstract

III–V photonic crystal (PhC) lasers with small footprints and low power consumption are potential ultra-compact and power-efficient light sources for future on-chip optical interconnects. Conventional PhC lasers fabricated by vertical epitaxy require suspended air-bridge structures and air holes etched through the gain medium, severely compromising mechanical resistance to external impacts and pumping efficiency. While bonding and regrowth can mitigate these issues, their fabrication complexity substantially increases process costs and hinders mass production. Here, we address these issues using selective lateral heteroepitaxy and demonstrate monolithically integrated III–V membrane PhC lasers on (001) silicon-on-insulator (SOI). By leveraging selective lateral heteroepitaxy and metal organic chemical vapor deposition (MOCVD), we achieved the growth of dislocation-free InP membranes on SOI wafers patterned in Si-photonics foundries. The unique III-V-on-insulator avoids the formation of air-suspended structures and significantly enhances the mechanical stability of the devices. We also precisely positioned the laterally grown InGaAs/InP quantum wells (QWs) at the center of the InP membrane to avoid etching air holes through the gain medium, thus eliminating surface recombination and drastically improving pumping efficiency. We fabricated near-infrared and telecom PhC lasers using laterally grown III–V membranes, and achieved room-temperature lasing at 910 nm and 1430 nm with low thresholds of 17.5 μJ/cm² and 5.7 μJ/cm², respectively. Our results establish a novel approach for fabricating PhC lasers and provide an elegant solution for monolithically integrated PhC lasers in next-generation optical interconnects.

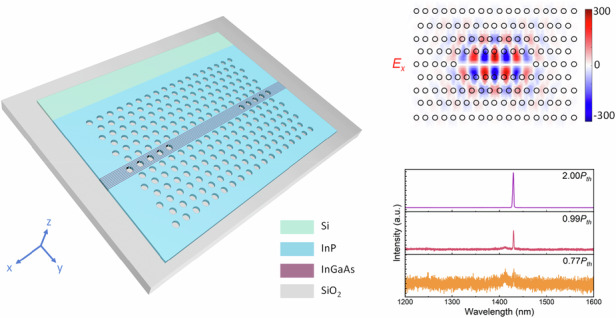

## Abstract (web summary)

We demonstrated monolithic III–V membrane photonic crystal (PhC) lasers on SOI using selective lateral heteroepitaxy, which enables high mechanical resistance to external impacts and high pumping efficiency within novel fabrication platform of monolithic III–V PhC lasers for Si photonics.

## Introduction

Amidst the rapid expansion of data traffic, photonic integrated circuits (PICs) have emerged as a transformative solution for high-speed data transmission and processing, enabling scalable deployments in optical interconnects^1^, computing^2^, and sensing^3^. In systems leveraging PICs for massive data communication, such as data centers^4^ and AI computing clusters^5^, the requirements for high-density integration and ultralow power consumption are becoming increasingly critical. Consequently, integrated light sources with smaller footprints and higher energy efficiency are essential to drive advancements in this field. Among various types of lasers, photonic crystal (PhC) lasers stand out due to their small mode volume, low threshold, and high quality factor^6^.

Since the proposition of PhC cavities^7^, III–V PhC lasers on native substrates have been developed with impressive performance^8–10^. The lasing thresholds of these devices have been reduced to the nanowatt or nanoampere levels^10,11^, and modulation bandwidths exceeding 100 GHz have been demonstrated^12^. While these achievements on native substrates are remarkable, the recent advancements in Si photonics have also enabled the integration of III–V PhC lasers on Si using heterogeneous integration or direct heteroepitaxy^13–16^. In most of the reported works, a vertically stacked structure based on low-index-contrast III–V material systems is typically employed, which necessitates air-suspended structures for vertical optical confinement and patterning air holes through the entire epi-layer for lateral optical confinement^17^. These features lead to the issues of mechanical stability and pumping efficiency. Firstly, the suspended structures formed by undercutting the sacrificial layer severely compromise the resistance to external mechanical impacts. This issue can be resolved through transferring the III–V membranes onto low-index substrates using heterogeneous integration approaches such as bonding or micro transfer printing^18–20^. Secondly, the vertically stacked structures with air holes etched through the gain medium result in severe non-radiative surface recombination and resultant low pumping efficiency. This issue can be addressed through forming buried heterostructures (BH) using selective regrowth^21^. The compact BH design centered within the PhC cavity avoids the etching of air holes through the active region. By combining BH and transfer techniques, lambda-scale embedded active-region photonic-crystal lasers have been integrated on Si substrates with an ultralow threshold of 31 μA^22^. However, this technique involves bonding, selective regrowth, and several doping steps. The significantly increased fabrication complexity leads to high costs, limits integration density, and hinders mass production.

**Fig. 1:**
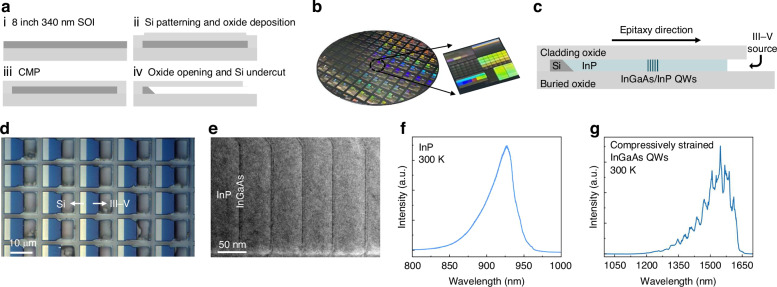
**Selective lateral heteroepitaxy of III–V membranes on SOI.** **a** Patterning process of the epitaxial templates for selective lateral heteroepitaxy. **b** Full view of an 8-inch commercial (001) SOI wafer with patterned design and a close-up photograph of a single die. **c** Schematic of selective lateral heteroepitaxy. **d** Optical microscope image of InP/InGaAs membranes laterally grown on SOI. **e** Cross-sectional TEM image of the compressive-strained InGaAs QWs. **f** PL spectrum of InP membranes. **g** PL spectrum of compressively strained InGaAs QWs

In this work, we address the above issues using selective lateral heteroepitaxy. Our approach fundamentally differs from conventional vertical epitaxy by forming III–V membranes immediately atop the buried oxide layer on SOI wafers^23–25^, as schematically illustrated in Fig. 1c. The resultant III–V-on-insulator exhibits distinct advantages for the fabrication of PhC lasers. Firstly, it offers an inherent high refractive index contrast between the III–V membrane and the silicon oxide, eliminating the need for suspended structures to achieve strong optical confinement. Secondly, the gain medium formed during selective lateral heteroepitaxy can be well controlled with precise width and position away from the subsequently etched airholes, eliminating surface recombination and thus improving pumping efficiency. Thirdly, selective lateral heteroepitaxy enables the monolithic PhC lasers on SOI wafers with high crystalline III–V membranes, offering the perspective of dense integration with high scalability and uniformity. In light of these distinct advantages, we report, to the best of our knowledge, the first monolithic III–V membrane PhC lasers laterally grown on (001) SOI substrates. Near-infrared (910 nm) InP and telecom (1430 nm) InP/InGaAs PhC lasers were fabricated from III–V membranes laterally grown on SOI and achieved room temperature lasing under optical pumping with low thresholds of 17.5 μJ/cm² and 5.7 μJ/cm², respectively.

## Results

### Selective lateral heteroepitaxy of III–V membranes on SOI

The selective lateral heteroepitaxy of III–V membranes on SOI in this work is based on the concept of lateral aspect ratio trapping^26^. Figure 1a depicts the patterning process of the epitaxial templates. Starting with an 8-inch 340-nm (001) SOI wafer, the Si layer is first patterned into segments with subsequent deposition of cladding oxide, followed by chemical mechanical polishing. The lateral oxide trenches with (111) Si facets are then formed by etching oxide openings at one end of the Si patterns and subsequent anisotropic wet etching of Si. The wafer and die of the templates fabricated in a commercial 8-inch Si-photonics foundry are presented in Fig. 1b. Figure 1c schematically illustrates the structural configuration and epitaxial process of selective lateral heteroepitaxy. InP preferentially nucleates on the monocrystalline Si seed over the amorphous SiO_2_ cladding layer. Inside the lateral trench, InP membrane evolves laterally towards the opening of the oxide trench with a length over 6 μm, and vertical InGaAs/InP quantum wells (QWs) can be embedded at desired positions along the InP membrane. The mature processing of Si photonics foundry guarantees smooth Si segments and results in InP membranes with atomic-flat surfaces. Details of the epitaxial process are described in ref. ^27^. The III–V membrane laterally grown within the oxide trench forms the unique III–V-on-insulator structure, featuring a high refractive index contrast without the need for additional processing steps. The air holes of the PhC cavity can be patterned on both sides of the laterally grown QWs, thus enabling spatial decoupling between the etched airhole sidewalls and the QW active region.

Figure 1d presents optical microscopy characterization of the laterally grown InP/InGaAs membranes on SOI. The III–V membranes demonstrate exceptional morphological uniformity with straight growth fronts over the epitaxial areas. Figure 1e displays cross-sectional transmission electron microscopy (TEM) analysis of compressively strained InGaAs QWs, revealing precisely engineered 6-nm In_0.7_Ga_0.3_As QWs sandwiched between 50-nm InP barrier layers. Room temperature photoluminescence (PL) spectra of the InP membranes and the embedded InGaAs QWs are plotted in Fig. 1f, g, respectively. The InP membranes show a narrow emission peak around 920 nm, and the InGaAs QWs manifest a peak wavelength around 1.55 μm, with full width at half maximum (FWHM) estimated to be 40 nm and 134 nm, respectively. The epitaxial results demonstrate that the lateral III–V membranes can be employed to fabricate both near-infrared and telecom-band PhC lasers.

### Near-infrared InP PhC lasers

**Fig. 2:**
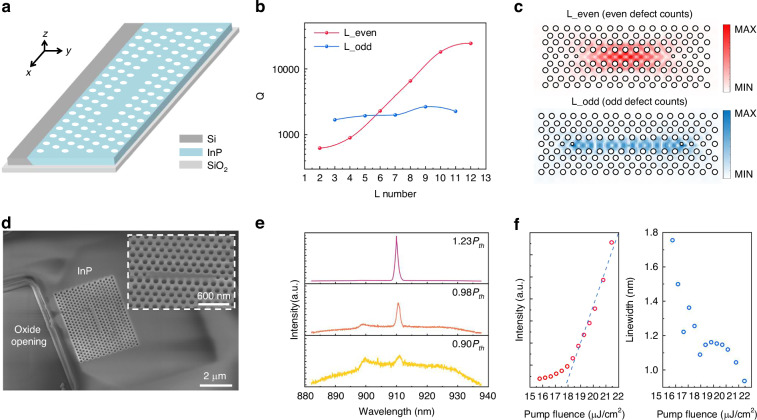
**Design, fabrication, and characterization of near-infrared InP PhC lasers.** **a** 3D architecture of the InP PhC laser. **b** Simulated Q-factor as a function of L number. **c** Electric field profiles of cavities with even and odd defect counts. **d** 70° tilted SEM images of the fabricated InP PhC laser. Inset: Zoom in SEM image of the cavity region. **e** Measured spectra under various pump powers. **f** Collected L–L curve (left) and linewidth evolution (right)

We first designed and fabricated near-infrared InP PhC lasers based on InP membranes laterally grown on SOI. Figure 2a depicts the device structure of the InP PhC laser. We adopted a triangular PhC lattice with L-type defect structure, which consists of several missing air holes along the Γ-K direction of the lattice. The cavity for InP PhC lasers is designed with lattice constant *a* = 210 nm, radius of the air hole *r* = 60 nm, and thickness of InP membranes *T* = 340 nm. To maintain robust mechanical resistance to external impacts and enhance heat dissipation capability^28^, the PhC lasers in this work do not undercut the buried oxide layer. The vertical asymmetry could affect the cavity mode profiles and cause degradation of the quality factor Q^29^, but a sufficiently high Q-factor can still be achieved through adjusting the cavity design. Three inner air holes on both sides of the cavity have a gradually smaller radius by 0.05*a* to reduce mutations of the envelope function of the mode profile and thereby increasing the Q-factor^30^, The dependence of Q-factor on the number of missing air holes (L number) is plotted in Fig. 2b. As the L number increases, PhC cavities with even-defect counts exhibit a gradual increase in Q-factor with maximum value exceeding 10⁴ followed by saturation, whereas those with odd-defect counts maintain Q-factor around 10³ without significant change. The electric field profiles of the cavity modes depicted in Fig. 2c explain the difference in Q-factor between even and odd L numbers. Together as even modes in terms of parity, the mode profile of the odd-defect-count cavity is more concentrated in the defect region, while that of the even-defect-count cavity is more dispersed. However, the odd cavity exhibits significant electric field discontinuities at both cavity edges, limiting further enhancement of the Q-factor, whereas the even cavity maintains uniform field distribution in these regions, enabling a higher Q-factor^31^. Given the uniform gain in the InP membranes minimizes modal gain differences between even and odd cavities, an L8 defect structure was adopted for the fabrication of near-infrared InP PhC lasers.

The fabrication of InP PhC lasers follows standard III–V semiconductor processing techniques^32^. The cladding oxide layer of the epitaxial templates was used as the hard mask for the dry etching of InP membranes after thinning down. The PhC cavities were then fabricated using electron beam lithography (EBL) and two dry etching processes for both the oxide mask and the InP membrane. Figure 2d presents the 70° tilted scanning electron microscopy (SEM) images of the fabricated InP PhC laser, and the inset provides a magnified view of the cavity region, illustrating the PhC cavity precisely centered within the epitaxial InP membrane. The fabricated lasers were characterized by room temperature PL under pulse excitation (750-nm wavelength, 80 MHz repetition frequency, <140 fs pulse width, 10 μm radius of the laser beam spot). Figure 2e plots the lasing spectra observed from the fabricated InP PhC laser. Below threshold, the emission manifests a broad spontaneous emission spectrum of InP with multiple cavity modes. With the pump power rising above threshold, the higher-order mode is suppressed, and the InP PhC laser exhibits single-mode lasing with a peak wavelength at 910 nm. The collected intensity (L–L) curve is shown on the left of Fig. 2f, from which a threshold pump power density of 17.5 μJ/cm^2^ can be extracted. For the measured data of InP PhC lasers, the lasing linewidth decreases when increasing the pump power and finally narrows to 0.94 nm, as shown in the right of Fig. 2f. The experimentally extracted Q-factor (*Q* = *λ*/Δ*λ*) of ~10³ is lower than the simulated value, with the degradation primarily attributed to non-vertical sidewalls in the etched air holes. Note that the emission wavelength of the PhC lasers can be readily tailored by adjusting the design parameters. The lasing performance of InP PhC lasers confirms the high crystalline quality of InP membranes laterally grown on SOI and the feasibility of the lateral epitaxial platform for scalable fabrication of PhC lasers.

### Telecom-band InP/InGaAs PhC lasers

**Fig. 3:**
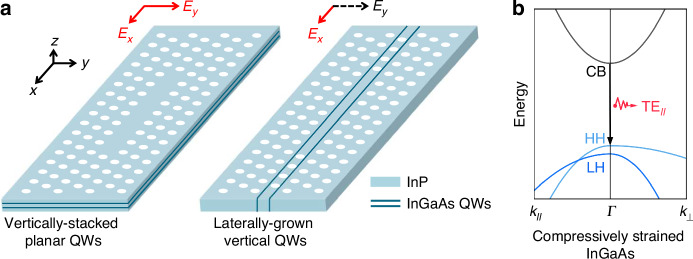
**Comparison of gain characteristics between vertically stacked planar QWs and laterally grown vertical QWs.** **a** PhC cavities based on vertically stacked planar QWs (left) and laterally grown vertical QWs (right). The red arrows indicate the electric field components that experience sufficient gain from the compressively strained QWs, and the black dashed arrows represent components with negligible gain contribution. **b** Energy band structure of compressively strained InGaAs with three primary energy bands: conduction band (CB), heavy-hole (HH) and light-hole (LH) valence bands

We then investigated the design and fabrication of telecom-band InP/InGaAs PhC lasers based on the InP membranes with embedded compressively strained InGaAs QWs. The lateral growth direction leads to the formation of vertical QWs, which are significantly different from conventional planar QWs in terms of designing PhC lasers. Figure 3a compares the two types of PhC cavity architectures: one based on vertically stacked planar QWs and our method with laterally grown vertical QWs. Correspondingly, for transverse electric (TE) mode, laterally grown vertical QWs exhibit different gain characteristics compared with vertically stacked planar QWs. Figure 3b illustrates the band structure of compressively strained InGaAs, where *Γ* denotes the center of the Brillouin zone, *k*_∥_ is in the plane of InGaAs, and *k*_*⊥*_ is perpendicular to the plane of InGaAs. The compressively strained InGaAs preferentially supports TE_∥_ modes by inducing biaxial strain that elevates the heavy-hole (HH) valence band above the light-hole (LH) band, thereby enhancing in-plane dipole transitions^33^. This band engineering yields a higher optical gain of in-plane polarized modes in terms of compressively strained InGaAs. Therefore, for the PhC cavity fabricated on vertically stacked planar QWs, both the *E*_*x*_ and *E*_*y*_ components lying within the plane of QWs can leverage sufficient optical gain. However, in PhC cavity with laterally grown vertical QWs, the *E*_*y*_ component no longer lies within the plane of the QWs, and thus the QWs only contribute optical gain for the *E*_*x*_ component. Based on the analyses above, we then focus primarily on the in-plane electric field component (*E*_*x*_) of the cavity modes in the gain design of the telecom PhC cavity.

**Fig. 4:**
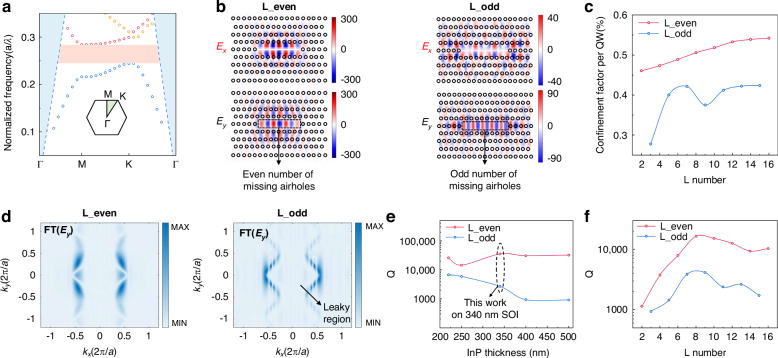
**Design of telecom InP/InGaAs membrane PhC lasers.** **a** Fundamental TE mode band diagram of the triangular lattice with the irreducible Brillouin zone. **b** Calculated *E*_*x*_ and *E*_*y*_ field profiles of cavities with even and odd defect counts. **c** Calculated confinement factors of each QW as a function of L number. **d** FT spectra of the *E*_*y*_ profiles for even and odd cavities, with the leaky region centered within the spectrum. **e** Simulated Q-factor as a function of InP thickness for even and odd cavities. **f** Simulated Q-factor as a function of L number

With the gain characteristics of laterally grown vertical QWs elucidated, we systematically designed the telecom PhC cavity, focusing on modal confinement and Q-factor. The PhC cavity used for telecom PhC lasers is a basic triangular lattice with *a* = 370 nm, *r* = 80 nm, and *T* = 340 nm. Figure 4a plots the calculated fundamental TE mode band diagram of the triangular lattice utilizing the 3D finite-difference time-domain (FDTD) method. In the photonic band diagram, the blue-shaded areas delineate the light cone, and the pink region corresponds to the photonic bandgap spanning normalized frequencies from 0.244 to 0.284. Figure 4b compares the electric field distributions of the *E*_*x*_ and *E*_*y*_ components for the fundamental mode excited by vertical QWs in L-cavities with even and odd L numbers. Considering the gain characteristics of laterally grown vertical QWs, the compressive-strained QWs used in this work are more suitable for an even-defect-count cavity with centered *E*_*x*_ confinement for sufficient optical gain. Calculated from the *E*_*x*_ distribution, the confinement factors of each QW are plotted as a function of the L number in Fig. 4c. The confinement factors of the even cavities are significantly larger than those of the odd cavities and exhibit a more consistent enhancement, which indicates that the even cavities possess superior modal confinement. It is crucial to clarify that even/odd in this work quantitatively describes the number of missing airholes in L-type PhC cavities, not the parity of distinct optical modes. From the perspective of parity, both of the modes excited in even and odd cavities belong to even modes.

Having addressed the gain design, we then proceed to investigate key factors influencing the Q-factor of the cavity. Figure 4d presents the Fourier-transform (FT) spectra of the *E*_*y*_ profiles for even and odd cavities^34^. The odd cavity exhibits significant components within the leaky region, which is one of the primary limiting factors for the achievable Q-factor^35^. For odd cavities, this leakage issue can be mitigated by adjusting the inner hole positions and dimensions^36^, whereas even cavities intrinsically avoid such a challenge due to their field distribution, as evidenced by the pristine FT spectrum showing negligible components within the leaky region. Increased vertical leakage in odd cavities results in significantly lower Q-factor compared to their even counterparts, with the disparity widening when the III–V membrane thickness increases, as shown in Fig. 4e. For III–V membranes with a thickness of 340 nm, the Q-factor of even cavities is one order of magnitude higher than that of odd cavities. Figure 4f plots the Q-factor as a function of the L number, revealing that even cavities consistently outperform their odd counterparts, with the L8 configuration achieving a maximum Q-factor exceeding 10⁴. Based on the comprehensive analyses above, the L8 defect structure was selected as the cavity design for the telecom PhC lasers, balancing sufficient optical gain, robust modal confinement, and high Q-factor.

**Fig. 5:**
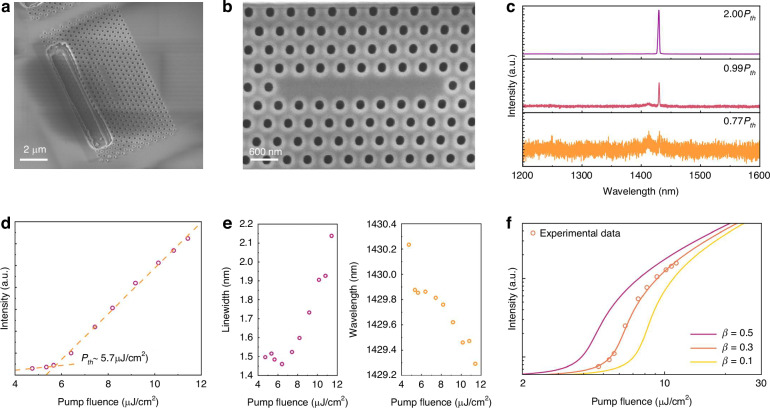
**Fabrication and characterization of telecom InP/InGaAs membrane PhC lasers.** **a** 70° tilted SEM image of the fabricated telecom PhC laser. **b** Zoom in SEM image of the PhC lattice. **c** Measured spectra under various pump powers. **d** Collected L–L curve with fitting lines. **e** Lasing linewidth (left) and wavelength (right) evolution. **f** Logarithmic L–L plot of the telecom PhC laser. The experimental data are plotted in circles, and the theoretically calculated results for various values of *β* are plotted in solid lines

The fabrication process for telecom PhC lasers closely resembles that of the near-infrared PhC lasers, with a critical requirement being that the defect region of the PhC cavity needs to be precisely aligned length-wise with the InGaAs QWs. Given that the total width of InGaAs QW layers is comparable with the width of *E*_*x*_ distribution, a slight misalignment between the position of QWs and the defect region of the PhC cavity would cause a significant decline of the modal gain. We used optical microscopy to approximately locate the QWs and incorporated variations of alignment widths between the cavity and QWs during fabrication to ensure that some devices could achieve precise alignment. Crucially, this alignment challenge does not fundamentally compromise the technical viability of this method, as both stable epitaxy processes and precise characterization techniques are readily available in semiconductor foundries. Routine semiconductor characterization techniques, such as focus ion beam milling-assisted SEM, could be employed to precisely align the PhC cavity with the QWs. Figure 5a presents the 70° tilted SEM image of the fabricated telecom PhC laser. The PhC cavity was precisely fabricated on the III–V membrane beneath the smooth oxide hard mask. Figure 5b displays the zoom-in SEM image of the PhC lattice, and the InGaAs QWs were positioned at the middle of the PhC lattice, where the best alignment with the cavity mode can be achieved.

Under the same pumping conditions as the near-infrared PhC lasers, the fabricated telecom PhC laser achieved single-mode lasing at 1.43 μm, with the lasing spectra shown in Fig. 5c. Below threshold, the spontaneous emission of InGaAs QWs is suppressed, revealing two coexisting cavity modes. As the pump power increases beyond threshold, the fundamental mode becomes dominant while the other is progressively suppressed, ultimately achieving single-mode lasing across the entire telecom band. Figure 5d plots the L–L curve, where the evidence of lasing with a clear upturn can be found, and a threshold pump power density of 5.7 μJ/cm^2^ can be extracted. Owing to the employment of QWs as the active region, the threshold of the telecom PhC lasers is lower than that of the near-infrared InP PhC lasers. The lasing linewidth and peak wavelength evolution are depicted in Fig. 5e. The lasing linewidth exhibits a non-monotonic dependence on pump power, achieving a minimum value of 1.46 nm near the threshold before broadening at higher excitation levels due to nonlinear phase noise^37^. The experimentally extracted Q-factor of ~10³ is significantly lower than the simulated value (>10⁴), which is similar to the case of near-infrared PhC lasers with non-ideal sidewalls. The measured lasing peak exhibits a blue shift with increasing pump power, which is mainly induced by the band-filling effect under pulsed excitation. Figure 5f plots the theoretical L–L curves calculated based on the rate equation model^38^. The best fit to the experimental data is observed with the spontaneous emission coupling factor *β* = 0.3. The fabricated telecom PhC laser demonstrates higher *β*-factor compared to previously reported quantum-well-based PhC lasers^38,39^, attributing to the effective decoupling between the active region and etched air holes, which substantially reduces the total non-radiative emission rate.

## Discussion

While we achieved pulsed lasing in telecom InP/InGaAs PhC lasers, lasing has not been observed under continuous-wave (CW) pumping. The insufficient modal gain and thermal degradation caused by compressively strained InGaAs QWs prevent CW operation. Consequently, CW operation could be achieved through increasing modal gain and thermal stability. Firstly, enhancing fabrication precision, including sidewall morphology and alignment accuracy, can significantly increase the experimental Q-factor and modal gain. Secondly, adjusting the cavity design for more concentrated *E*_*x*_ distribution can enhance modal gain through increasing confinement factor as well as fabrication tolerance. Heterostructure nanocavity with centered *E*_*x*_ distribution along the QW direction is an ideal cavity for future devices with a lower threshold^40^. Finally, adjusting the QW active region, including composition and strain, can improve thermal stability. Employing either InGaAsP QWs or tensile-strained QWs increases the conduction band offset, which notably suppresses carrier leakage at elevated temperatures.

Besides enhancing the mechanical resistance to external impacts and pumping efficiency of PhC lasers, selective lateral heteroepitaxy also exhibits significant potential for enabling electrically pumped PhC lasers and high-efficiency coupling with Si waveguides. Firstly, a planar p-i-n structure can be achieved through in-situ doping during selective lateral heteroepitaxy in a single growth step^41,42^. The lateral current injection architecture enables substantial suppression of metal-induced absorption loss while eliminating the need for multi-step doping processes employed in conventional vertical epitaxy^15,22^, thereby significantly reducing fabrication complexity. Secondly, selective lateral heteroepitaxy enables intimate co-planar placement of epitaxial III–V membrane and Si device layer. High-efficiency light couplers as well as waveguides can be fabricated along with the epitaxial templates in a simple manner^43,44^. The compact butt couplers enable efficient light coupling into Si waveguides, paving the way for robust optical interfacing for fully integrated photonic systems on SOI. In addition to PhC cavities^45,46^, the III–V membrane platform enabled by selective lateral heteroepitaxy could also be employed to fabricate a variety of micro/nano laser cavities, such as Dirac-vortex topological cavity^47^ and extreme dielectric confinement cavity^48^, supporting both edge-emitting and vertical emission configurations.

The monolithic membrane PhC lasers grown by selective lateral heteroepitaxy offer additional advantages of simplified fabrication and significantly reduced processing time and costs. Firstly, the fabrication of epitaxial templates is performed in Si photonics foundries, enabling substantial reductions in both process cycle time and costs. Secondly, selective growth at predefined regions via MOCVD not only consumes minimal III–V precursors but also effectively reduces the growth time, drastically cutting epitaxial costs and time expenditure. Thirdly, compared with conventional methods of fabricating PhC lasers using undercutting sacrificial layers or transferring onto low-index substrates, our method significantly simplifies the fabrication process while maintaining the advantages of high mechanical resistance to external impacts and pumping efficiency.

In conclusion, we demonstrated the monolithic integration of near-infrared and telecom III–V membrane PhC lasers on (001) SOI substrates through selective lateral heteroepitaxy. Our approach not only exhibits high mechanical resistance to external impacts through avoiding the formation of air-suspended structures but also manifests high pumping efficiency through spatially decoupling the gain medium from the etched air holes. Room-temperature lasing was demonstrated from both near-infrared InP and telecom InP/InGaAs membrane PhC lasers, with low thresholds of 17.5 μJ/cm² and 5.7 μJ/cm² under optical pumping. Our work provides a novel solution for fabricating PhC lasers and marks an important step towards electrically pumped PhC lasers directly grown on SOI in the near future.

## Materials and methods

### III–V membrane growth

We used commercially patterned (001) SOI substrates as epitaxial templates for selective lateral heteroepitaxy of III–V membranes. The fabrication process for the epitaxial templates has been detailed in the selective lateral heteroepitaxy part of this work. Before growth, the sample was cleaned by the standard RCA (NH_4_OH:H_2_O_2_:H_2_O = 1:1:5) process for 10 minutes. The sample was subsequently etched in a 1% hydrofluoric acid (HF) solution for 20 seconds to remove the native oxide layer from the Si seed, followed by thorough rinsing with deionized water. Then we performed selective lateral heteroepitaxy of III–V membranes in an AIXTRON CS18271 MOCVD system. The InP membranes were laterally grown employing a unique three-step growth scheme, and the InGaAs/InP QWs were embedded at desired positions, with details of the epitaxial process described in ref. ^27^.

### PhC cavity simulation

The optical simulation of the PhC cavity was calculated using the three-dimensional FDTD method. The simulated model incorporated the lateral InP membrane immediately atop the buried oxide layer with adjacent Si seed and top oxide hard mask. To estimate the Q factor, time-domain simulation data is first collected by a group of time monitors. All resonant modes within the specified frequency range are subsequently identified, with the Q factor for each resonance calculated from the slope of the decaying envelope. Beyond Q factor, additional mode analyses including field distributions, confinement factors, and Fourier-transformed spectra are based on the simulation data from frequency-domain power monitors. Band structure calculations employ eigenfrequency extraction through Fourier analysis of time-domain field data under Bloch-periodic boundary conditions, sampling wavevectors *k* across the Brillouin zone.

### Device fabrication and characterization

The fabrication process of the near-infrared InP and telecom InP/InGaAs membrane PhC lasers was categorized into four steps. Firstly, the cladding oxide layer of the epitaxial templates was thinned down to 200 nm as the hard mask using reactive ion etching (RIE). Secondly, the PhC patterns were defined using EBL in a 400 nm ARP6200 electron beam resist spin-coated on the surface of the hard mask. Afterwards, the hard mask was etched in RIE to transfer the PhC patterns, and the electron beam resist was subsequently removed using inductively coupled plasma RIE (ICP-RIE) with O_2_ plasma. Finally, the air holes on InP membranes with a thickness of 340 nm were etched in ICP-RIE based on a mixture of CH_4_, H_2_, and O_2_.

The fabricated near-infrared and telecom PhC lasers were characterized in a confocal micro-PL system at room temperature. A 750-nm femtosecond-pulsed laser was used to excite the devices using a 20× objective with a beam spot diameter estimated to be ∼20 µm. The emitted photons were collected using the same objective, and the spectrum characterization was analyzed by a monochromator with a cooled InGaAs detector. The pump power density was calculated based on the measured optical power, pulse repetition rate, and area of the laser beam spot.

## Data Availability

The data that support the findings of this study are available from the corresponding author upon reasonable request.

## References

[1] Shekhar, S. et al. Roadmapping the next generation of silicon photonics. *Nat. Commun.* **15**, 751 (2024). 38272873 10.1038/s41467-024-44750-0 PMC10811194

[2] Shastri, B. J. et al. Photonics for artificial intelligence and neuromorphic computing. *Nat. Photonics* **15**, 102–114 (2021).

[3] Passaro, V. M. N. et al. Recent advances in integrated photonic sensors. *Sensors* **12**, 15558–15598 (2012). 23202223 10.3390/s121115558 PMC3522976

[4] Shi, Y. C. et al. Silicon photonics for high-capacity data communications. *Photonics Res.* **10**, A106–A134 (2022).

[5] Biasi, S. et al. Photonic neural networks based on integrated silicon microresonators. *Intell. Comput.* **3**, 0067 (2024).

[6] Iadanza, S. et al. Photonic crystal lasers: from photonic crystal surface emitting lasers (PCSELs) to hybrid external cavity lasers (HECLs) and topological PhC lasers [Invited]. *Opt. Mater. Express* **11**, 3245–3274 (2021).

[7] Yablonovitch, E. Inhibited spontaneous emission in solid-state physics and electronics. *Phys. Rev. Lett.* **58**, 2059–2062 (1987). 10034639 10.1103/PhysRevLett.58.2059

[8] Nomura, M. et al. Room temperature continuous-wave lasing in photonic crystal nanocavity. *Opt. Express* **14**, 6308–6315 (2006). 19516806 10.1364/oe.14.006308

[9] Nomura, M. et al. Photonic crystal nanocavity laser with a single quantum dot gain. *Opt. Express* **17**, 15975–15982 (2009). 19724596 10.1364/OE.17.015975

[10] Ellis, B. et al. Ultralow-threshold electrically pumped quantum-dot photonic-crystal nanocavity laser. *Nat. Photonics* **5**, 297–300 (2011).

[11] Strauf, S. et al. Self-tuned quantum dot gain in photonic crystal lasers. *Phys. Rev. Lett.* **96**, 127404 (2006). 16605958 10.1103/PhysRevLett.96.127404

[12] Altug, H., Englund, D. & Vučković, J. Ultrafast photonic crystal nanocavity laser. *Nat. Phys.* **2**, 484–488 (2006).

[13] Zhou, T. J. et al. Continuous-wave quantum dot photonic crystal lasers grown on on-axis Si (001). *Nat. Commun.* **11**, 977 (2020). 32080180 10.1038/s41467-020-14736-9 PMC7033092

[14] Tanabe, K. et al. Room temperature continuous wave operation of InAs/GaAs quantum dot photonic crystal nanocavity laser on silicon substrate. *Opt. Express* **17**, 7036–7042 (2009). 19399078 10.1364/oe.17.007036

[15] Dimopoulos, E. et al. Electrically-driven photonic crystal lasers with ultra-low threshold. *Laser Photonics Rev.* **16**, 2200109 (2022).

[16] Mauthe, S. et al. Hybrid III–V silicon photonic crystal cavity emitting at telecom wavelengths. *Nano Lett.* **20**, 8768–8772 (2020). 33216555 10.1021/acs.nanolett.0c03634

[17] Painter, O. et al. Two-dimensional photonic band-gap defect mode laser. *Science* **284**, 1819–1821 (1999). 10364550 10.1126/science.284.5421.1819

[18] Crosnier, G. et al. Hybrid indium phosphide-on-silicon nanolaser diode. *Nat. Photonics* **11**, 297–300 (2017).

[19] Lu, T. W. et al. III-V photonic crystal nanobeam lasers side-coupled to silicon waveguide by transfer printing. *J. Light Technol.* **43**, 4314–4321 (2025).

[20] Park, B. J. et al. Minimal-gain-printed silicon nanolaser. *Sci. Adv.* **10**, eadl1548 (2024). 39292779 10.1126/sciadv.adl1548 PMC11409962

[21] Matsuo, S. et al. High-speed ultracompact buried heterostructure photonic-crystal laser with 13 fJ of energy consumed per bit transmitted. *Nat. Photonics* **4**, 648–654 (2010).

[22] Takeda, K. et al. Heterogeneously integrated photonic-crystal lasers on silicon for on/off chip optical interconnects. *Opt. Express* **23**, 702 (2015). 25835830 10.1364/OE.23.000702

[23] Han, Y., Xue, Y. & Lau, K. M. Selective lateral epitaxy of dislocation-free InP on silicon-on-insulator. *Appl. Phys. Lett.* **114**, 192105 (2019).

[24] Yan, Z. et al. Lateral tunnel epitaxy of GaAs in lithographically defined cavities on 220 nm silicon-on-insulator. *Cryst. Growth Des.* **23**, 7821–7828 (2023). 10.1021/acs.cgd.3c00633 PMC10626574 37937193

[25] Scherrer, M. et al. In-plane monolithic integration of scaled III-V photonic devices. *Appl. Sci.* **11**, 1887 (2021).

[26] Yan, Z. et al. A monolithic InP/SOI platform for integrated photonics. *Light Sci. Appl.* **10**, 200 (2021). 34565795 10.1038/s41377-021-00636-0 PMC8473568

[27] Fu, D. H. et al. Buried InGaAs/InP quantum wells selectively grown on SOI for lateral membrane laser diodes. *Appl. Phys. Lett.* **124**, 081102 (2024).

[28] Monat, C. et al. Two-dimensional hexagonal-shaped microcavities formed in a two-dimensional photonic crystal on an InP membrane. *J. Appl. Phys.* **93**, 23–31 (2003).

[29] Tanaka, Y. et al. Investigation of point-defect cavity formed in two-dimensional photonic crystal slab with one-sided dielectric cladding. *Appl. Phys. Lett.* **88**, 011112 (2006).

[30] Akahane, Y. et al. Fine-tuned high-Q photonic-crystal nanocavity. *Opt. Express* **13**, 1202–1214 (2005). 19494990 10.1364/opex.13.001202

[31] Akahane, Y. et al. Investigation of high-*Q* channel drop filters using donor-type defects in two-dimensional photonic crystal slabs. *Appl. Phys. Lett.* **83**, 1512–1514 (2003).

[32] Dranczewski, J. et al. Plasma etching for fabrication of complex nanophotonic lasers from bonded InP semiconductor layers. *Micro Nano Eng.* **19**, 100196 (2023).

[33] Adams, A. R. Strained-layer quantum-well lasers. *IEEE J. Sel. Top. Quantum Electron.* **17**, 1364–1373 (2011).

[34] Srinivasan, K. & Painter, O. Momentum space design of high-Q photonic crystal optical cavities. *Opt. Express* **10**, 670–684 (2002). 19451920 10.1364/oe.10.000670

[35] Kim, S. H. et al. Characteristics of a stick waveguide resonator in a two-dimensional photonic crystal slab. *J. Appl. Phys.* **95**, 411–416 (2004).

[36] Akahane, Y. et al. High-*Q* photonic nanocavity in a two-dimensional photonic crystal. *Nature* **425**, 944–947 (2003). 14586465 10.1038/nature02063

[37] Henry, C. Theory of the linewidth of semiconductor lasers. *IEEE J. Quantum Electron.* **18**, 259–264 (1982).

[38] Altug, H. & Vučković, J. Photonic crystal nanocavity array laser. *Opt. Express* **13**, 8819–8828 (2005). 19498914 10.1364/opex.13.008819

[39] Ryu, H. Y. et al. Large spontaneous emission factor (> 0.1) in the photonic crystal monopole-mode laser. *Appl. Phys. Lett.* **84**, 1067–1069 (2004).

[40] Takahashi, Y. et al. A micrometre-scale Raman silicon laser with a microwatt threshold. *Nature* **498**, 470–474 (2013). 23803846 10.1038/nature12237

[41] Xue, Y. et al. High-speed and low dark current silicon-waveguide-coupled III-V photodetectors selectively grown on SOI. *Optica* **9**, 1219–1226 (2022).

[42] Mauthe, S. et al. High-speed III-V nanowire photodetector monolithically integrated on Si. *Nat. Commun.* **11**, 4565 (2020). 32917898 10.1038/s41467-020-18374-z PMC7486389

[43] Ren, Z. J. & Han, Y. Compact light couplers for lateral III–V membrane devices grown on SOI platforms. *Opt. Lett.* **49**, 2685–2688 (2024). 38748136 10.1364/OL.524405

[44] Wen, P. Y. et al. Waveguide coupled III-V photodiodes monolithically integrated on Si. *Nat. Commun.* **13**, 909 (2022). 35177604 10.1038/s41467-022-28502-6 PMC8854727

[45] Portalupi, S. L. et al. Planar photonic crystal cavities with far-field optimization for high coupling efficiency and quality factor. *Opt. Express* **18**, 16064–16073 (2010). 20720991 10.1364/OE.18.016064

[46] Kim, S. H., Kim, S. K. & Lee, Y. H. Vertical beaming of wavelength-scale photonic crystal resonators. *Phys. Rev. B* **73**, 235117 (2006).

[47] Gao, X. M. et al. Dirac-vortex topological cavities. *Nat. Nanotechnol.* **15**, 1012–1018 (2020). 33077965 10.1038/s41565-020-0773-7

[48] Xiong, M. et al. A nanolaser with extreme dielectric confinement. Print at 10.48550/arXiv.2412.02844 (2024). 10.1126/sciadv.adx3865 PMC12710687 41406223

